# Deregulated expression of cytoskeleton related genes in the spinal cord and sciatic nerve of presymptomatic SOD1^G93A^ Amyotrophic Lateral Sclerosis mouse model

**DOI:** 10.3389/fncel.2014.00148

**Published:** 2014-05-26

**Authors:** Jessica R. Maximino, Gabriela P. de Oliveira, Chrystian J. Alves, Gerson Chadi

**Affiliations:** Department of Neurology, Neuroregeneration Research Center, University of São Paulo School of MedicineSão Paulo, Brazil

**Keywords:** ALS, SOD1^G93A^, pre-symptomatic, spinal cord, sciatic nerve, Kif1b microarray

## Abstract

Early molecular events related to cytoskeleton are poorly described in Amyotrophic Lateral Sclerosis (ALS), especially in the Schwann cell (SC), which offers strong trophic support to motor neurons. Database for Annotation, Visualization and Integrated Discovery (DAVID) tool identified cytoskeleton-related genes by employing the Cellular Component Ontology (CCO) in a large gene profiling of lumbar spinal cord and sciatic nerve of presymptomatic SOD1^G93A^ mice. One and five CCO terms related to cytoskeleton were described from the spinal cord deregulated genes of 40 days (actin cytoskeleton) and 80 days (microtubule cytoskeleton, cytoskeleton part, actin cytoskeleton, neurofilament cytoskeleton, and cytoskeleton) old transgene mice, respectively. Also, four terms were depicted from the deregulated genes of sciatic nerve of 60 days old transgenes (actin cytoskeleton, cytoskeleton part, microtubule cytoskeleton and cytoskeleton). *Kif1b* was the unique deregulated gene in more than one studied region or presymptomatic age. The expression of *Kif1b* [quantitative polymerase chain reaction (qPCR)] elevated in the lumbar spinal cord (40 days old) and decreased in the sciatic nerve (60 days old) of presymptomatic ALS mice, results that were in line to microarray findings. Upregulation (24.8 fold) of *Kif1b* was seen in laser microdissected enriched immunolabeled motor neurons from the spinal cord of 40 days old presymptomatic SOD1^G93A^ mice. Furthermore, *Kif1b* was dowregulated in the sciatic nerve Schwann cells of presymptomatic ALS mice (60 days old) that were enriched by means of cell microdissection (6.35 fold), cell sorting (3.53 fold), and primary culture (2.70 fold) technologies. The gene regulation of cytoskeleton molecules is an important occurrence in motor neurons and Schwann cells in presymptomatic stages of ALS and may be relevant in the dying back mechanisms of neuronal death. Furthermore, a differential regulation of *Kif1b* in the spinal cord and sciatic nerve cells emerged as key event in ALS.

## Introduction

Amyotrophic Lateral Sclerosis (ALS) is a progressive, rapid and fatal neurodegenerative disease that affects motor neurons of the spinal cord, brainstem, and cerebral cortex (Tripathi and Al-Chalabi, 2008). The mortality is often due to a respiratory failure (Shaw et al., 2001).

ALS pathogenesis is still unknown. Nevertheless, the mechanisms underlying neurodegeneration in ALS seem multifactorial and take place in neurons and non-neuronal cells (Boillee et al., 2006a,b; Yamanaka et al., 2008; Wang et al., 2013). Recent analyses have showed the involvement of cytoskeleton, leading a disruption of intracellular function, and intercellular communication, with relevance to the triggering of motor neuron death (Guipponi et al., 2010). In fact, those events are especially important to motor neurons, highly polarized cells that establish contact with their target and surrounding Schwann cells through long axons.

The steady bidirectional flux of molecules and organelles in the motor neuron axons is necessary for cell survival and maintenance (Liu et al., 2013; Vinsant et al., 2013). In this context, cytoskeleton impairments might account for the described ALS mechanisms as regarding axonal/mitochondrial alteration, signaling endosome dysfunction, protein aggregation and apoptosis (Boillee et al., 2006a,b; Ferraiuolo et al., 2011; Kiernan et al., 2011; Usuki et al., 2012).

The presence of Schwann cell-expressing the distress biomarker ATF-3 in spinal nerves (Malaspina et al., 2010) before symptom onset suggests the contribution of those cells to ALS pathogenesis (Keller et al., 2009). Remarkably, axonal retraction and motor neuron disconnection from neuromuscular joints are ALS early events (Fischer et al., 2004; Parkhouse et al., 2008) that seem to be induced by Schwann cell mechanisms (Vinsant et al., 2013). For instance, distal Schwann cells produce semaphorin 3, a chemorepellent molecule for terminal axons (De Winter et al., 2006). Furthermore, the expression of the glial intermediate filament protein GFAP in Schwann cells of the peripheral nerve implies a dynamic alteration of cytoskeleton and turnover of myelin sheath (Hanyu et al., 1982). Moreover, an accumulation of iNOS immunoreactivity at the paranodal regions of Schwann cell myelin sheaths of peripheral nerves of presymptomatic ALS mice gives additional evidence for the impaired paracrine mechanisms between motor neuron and Schwann cell (Chen et al., 2010). Thus, it should be considered that the early peripheral events related to cytoskeleton of motor neurons and Schwann cells may contribute to neuronal dying back via disruption of peripheral neurotrophic stimuli (Keller et al., 2009; Dadon-Nachum et al., 2011; Gould and Kendall, 2011; Gould and Oppenheim, 2011; Liu et al., 2013).

As a short lasting disease, the challenge on ALS investigation is the employment of an adequate experimental model to evaluate presymptomatic mechanisms triggering motor neuron death. With this regard, it is known that transgene mice expressing human mutant copper/zinc superoxide dismutase 1 (SOD1^G93A^) develop clinical and pathological features similar to those seen in human ALS and are considered an excellent model to study the pathogenic mechanisms of the disease (Gama Sosa et al., 2012). The model is particularly useful to evaluate the events related to motor neuron degeneration prior neurological symptoms (Alves et al., 2011).

Large-scale microarray-based gene expression has been trying to identify new molecular cues potentially involved in the ALS pathogenesis both in animal models and postmortem tissue (Olsen et al., 2001; Hensley et al., 2002; Yoshihara et al., 2002; Dangond et al., 2004; Perrin et al., 2005; Ferraiuolo et al., 2007, 2009; Fukada et al., 2007; Lobsiger et al., 2007; Vargas et al., 2008; Kudo et al., 2010; Boutahar et al., 2011; Cooper-Knock et al., 2012). However, there is a lack of investigation on the analysis of cytoskeleton-related gene profiling. The evaluation of deregulated genes in specific enriched cells obtained by *in vitro* purification, single cell laser microdissection or cell sorting might contribute to refine the alterations of gene expression-related to cytoskeleton molecules on specific cells of peripheral motor neuron unit.

By means of a high-density oligonucleotide microarray-linked to specific tools capable to identify cellular components, the aim of this work was to identify the regulation of cytoskeleton-related genes in the presymptomatic stage in the spinal cord and sciatic nerve of the SOD1^G93A^ mouse model. The work has also evaluated the modulation of *Kif1b* in the enriched spinal cord motor neurons and sciatic nerve Schwann cells.

## Materials and methods

### Animal and tissue sample

Transgene SOD1^G93A^ mice (The Jackson Laboratory, Bar Harbor, ME, USA) were crossbred and the colony was maintained in a specific pathogen-free environment of the animal facility of University of São Paulo Medical School (São Paulo, Brazil) as described previously (Gurney, 1994; Scorisa et al., 2010; Alves et al., 2011). Animals were kept under controlled temperature and humidity conditions with a standardized light–dark cycle (lights on at 7.00 a.m. and off at 7.00 p.m.) and free access to food pellets and tap water. Mice were genotyped by PCR amplification of DNA extracted from their tails in order to identify the SOD1 mutation (Gurney, 1994; Scorisa et al., 2010; Alves et al., 2011). The study was conducted under protocols approved by the Animal Care and Use of Ethic Committee at University of São Paulo and in accordance to the Guide for the Care and Use of Laboratory Animals adopted by the National Institutes of Health.

Forty, 60, and 80 days old presymptomatic specific pathogen-free male SOD1^G93A^ mice and their age-paired wild-type controls (20–25 g body weight) were used in the experiments. No motor neuron death was seen in those animal ages (Alves et al., 2011) so that they were chosen for the present presymptomatic analyses. Animals were killed by decapitation. Lumbar spinal cords (40 and 80 days old mice) and sciatic nerves (60 days old mice) were removed, frozen, and stored at −80°C until use. Four-five animals per group were used in the microarray experiments. The quantitative polymerase chain reaction (qPCR) analyses of lumbar spinal cords (40 days old mice), and sciatic nerves (60 days old mice) as well as of enriched cells samples (60 days old mice) employed four mice of each transgene and wild-type groups.

### RNA isolation and microarray experiments

The procedures of microarray experiments and statistical analysis of the mouse spinal cords were described in our previous publication which has employed a Whole Mouse Genome Oligo 4 × 44 K microarray platform (Agilent Technologies, USA) (De Oliveira et al., 2013). Regarding the sciatic nerve samples, total RNA was isolated using the Miniprep kit (Zymo, USA). The procedure was performed according to the manufacturer's instructions. The quantity and integrity of RNA were determined by spectrophotometer (Nanodrop, Thermo Scientific, USA) and microfluidics-based electrophoresis (Agilent 2100 Bioanalyzer, Agilent Technologies, USA), respectively. RNA samples with OD 260/280 of approximately 2.0 and RIN >7.0 were used for microarray experiments and qPCR. A pool of RNAs from neonatal organs (heart, kidney, liver) was used as reference sample. A representative electropherogram from Bioanalyzer evaluation of RNA integrity of the sciatic nerve samples is shown in the supplementary material (Figure S1).

In the case of sciatic nerve analysis, RNAs of samples (25 ng) and reference (100 ng) were reverse transcribed by the Low-input RNA Linear Amplification kit and then transcribed to Cy3-labelled (samples) or Cy5-labelled (reference) according to the manufacturer's instructions (Agilent Technologies, USA) and to previous descriptions (De Oliveira et al., 2013, 2014).

A total of 300 ng of Cy3-labelled cRNA was hybridized together with the same amount of Cy5-labelled reference to Whole Mouse Genome Oligo 8 × 60 K. After an overnight hybridization at 65°C, the slides were washed and treated with a Stabilizing and Drying Solution (Agilent Technologies, USA) and scanned (Agilent Microarray Scanner). All steps were performed according to the manufacturer's instructions (Agilent Technologies, USA).

The raw data from hybridizations and experimental conditions are available on the Gene Expression Omnibus website under accession numbers GSE50642 (spinal cord analysis, according to De Oliveira et al., 2013) and GSE56926 (sciatic nerve analysis).

### Microarray analysis

Raw image data were converted to numerical data using the Agilent Feature Extraction Software, version 9.1.3.1 (spinal cord) (De Oliveira et al., 2013) and version 11.0.1.1 (sciatic nerve).

Microarrays without enough quality were taken out from further analyses, and the study proceeded with four samples for each group in the both studied regions. As already described for spinal cord in our previous study (De Oliveira et al., 2013), sciatic nerve microarray raw data (.txt files) were transferred to R v. 3.0.1 software (Team RDC, 2012) and analyzed with the Bioconductor (Gentleman et al., 2004) package limma (Smyth, 2005). Finally, the probes were tested for differential expression using a linear model followed by Bayes moderated t-test (Smyth, 2005) for the comparisons of interest. *P*-values < 0.05 were accepted as differentially expressed genes.

### Complementary DNA microarray data analysis

The Database for Annotation, Visualization and Integrated Discovery (DAVID) v6.7b functional tool (http://david.abcc.ncifcrf.gov/) (Huang Da et al., 2008) was used to identify genes related to cytoskeleton through the Gene Ontology (GO) annotation database. DAVID analysis focused in the Cellular Component Ontology (CCO). The analysis was conducted on the lists containing the up-regulated and down-regulated genes for each experimental group. High stringency (EASE score set to 0.05) parameters were selected to improve confidence on the terms to be pointed as enriched. Cellular component terms related to cytoskeleton gene lists were then organized. The BioVenn tool (http://www.cmbi.ru.nl/cdd/biovenn/) (Hulsen et al., 2008) was used to identify common and exclusively expressed genes between groups.

### Laser capture microdissection of motor neurons from spinal cord

**Figure 1:**
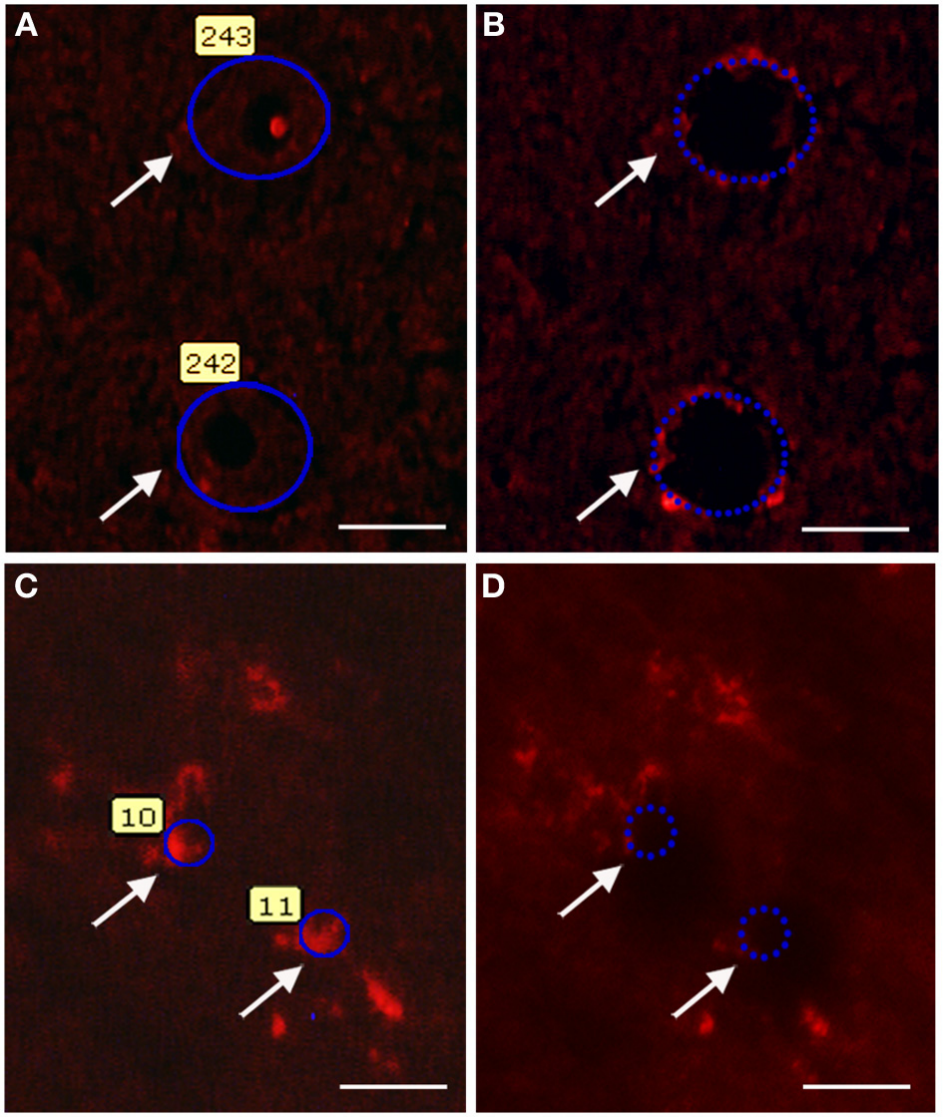
**Photomicrographs illustrating motor neuron (A,B) and Schwann cell (C,D) laser microdissection process**. The quick ChAT and S100β immunofluorescence procedures allow recognizing the motor neuron **(A,B)** and Schwann cell **(C,D)** profiles (arrows). Profiles were then selected for microdissection **(A,C)**. After laser firing and microdissection, selected cell profiles (arrows) can no longer be visualized in the tissue **(B,D)**. Scale bars: 20 μm.

Immunolabeled motor neurons of lumbar mouse spinal cord (SOD1^G93A^ and wild-type groups) were microdissected as described previously (De Oliveira et al., 2009, 2013). Spinal cord sections were rinsed for 3 min in phosphate buffered saline (PBS) containing 3% Triton X-100 (Sigma, USA) and then incubated overnight with a polyclonal goat anti-choline acetyltransferase (ChAT, 1:100; Abcam, USA) diluted in 0.3% Triton X-100 containing 1% bovine serum albumin (BSA; Sigma, USA), 1 mM dithiothreitol (DTT; Invitrogen, CAN), and 0.1 U/μl RNAse inhibitor (Invitrogen, CAN). Sections were then washed in PBS (3 × 15 s) and then incubated for 1 h in the dark and at room temperature with an Alexa 594-conjugated donkey anti-goat antibody (Invitrogen, USA) diluted (1:100) in the solution described above. Sections were rinsed carefully three times with PBS for 15 s and immediately submitted to single cell laser microdissection procedures. The ChAT immunofluorescence profiles for specific identification of motor neurons in the microdissection procedure are illustrated in Figures 1A,B.

About 100 motor neurons were isolated from each lumbar spinal cord using P.A.L.M. Microlaser Technologies (Zeiss). RNA was extracted from the microdissected motor neurons using the PicoPure RNA isolation kit (Arcturus, USA). Linear amplification of RNA was performed following Eberwine's procedure (Van Gelder et al., 1990) using the RiboampHSplus kit (Arcturus, USA) according to the manufacturer's protocol. The quantity and quality of the amplified RNA were analyzed as described above. Laser microdissected motor neuron samples were submitted to PCRs for verification of sample enrichment and the results are shown in the supplementary material (Figure S2).

### Laser capture microdissection of schwann cells from sciatic nerve

Sciatic nerve of 60 days old mice (SOD1^G93A^ and wild-type groups) were rapidly removed and frozen in ice cold isopentane at −45°C and stored at −80°C until use. The labeling procedure was performed according to adaptation of a previous description (De Oliveira et al., 2009, 2013). Taw-mounted mouse sciatic nerve sections (5 μm) were rapidly defrosted for 30 s and fixed with ice-cold acetone, for 3 min. Sections were then rinsed (3 min) in PBS containing 3% Triton X-100 and incubated with a polyclonal rabbit anti-S100β antibody (1:200; Dako, USA) diluted in PBS containing 0.3% Triton X-100 (Sigma, USA) and 1% BSA (Sigma, USA) for 5 min. Sections were washed in PBS (3 × 15 s) and then incubated (5 min) in the dark and at room temperature with a texas red-conjugated goat anti-rabbit antibody (Jackson ImmunoResearch Laboratories, USA) diluted (1:50) in the solution described above. Sections were then rinsed carefully three times with PBS for 15 s and immediately submitted to single cell laser microdissection procedures. Schwann cell S100β immunofluorescence profiles identified in the microdissection procedure are illustrated in Figures 1C,D.

About 200 Schwann cells were isolated from each sciatic nerve using the P.A.L.M. Microlaser Technologies. The RNA was extracted from the cells and amplified as described above. The quantity and quality of amplified RNA were analyzed as described above. Laser microdissected Schwann cell profiles were also submitted to PCRs for verification of sample enrichment. The detailed protocol and results are shown in the supplementary material (Figure S3).

### Flow cytometry sorting schwann cells

Schwann cells were isolated by means of flow cytometry sorting from the sciatic nerve explants of 60 days old SOD1^G93A^ mice and their age-paired wild-type controls. Briefly, animals were deeply anaesthetized with sodium pentobarbital 3% (100 mg/kg, ip) and their sciatic nerves were dissected under aseptic conditions. Nerves were then placed in 60 mm dishes containing Leibovitz-15 medium (Gibco, USA), divested of their epineurial sheaths and chopped into 1 mm pieces. The fragments were then transferred to new 60 mm dishes containing D-10 culture medium [composed by DMEM (Gibco, USA) supplemented with 10% fetal bovine serum and 1% penicillin/streptomycin (Sigma, USA)] and were maintained there in 5% CO_2_ at 37°C for 5 days. The sciatic nerve fragments were then transferred to 30 mm dishes containing 2.5 ml Hanks' Balanced Salt solution (Sigma, USA), 0.05% trypsin (Gibco, USA), and 1 mg collagenase (Worthington, USA). The fragments were kept in that solution for 2 h in 5% CO_2_ at 37°C. Tissue fragments were washed with D-10 and dissociated by trituration through a 200 μl-pipette and a 19-gauge sterile needle. The suspension was centrifuged at 1500 rpm for 5 min at 4°C and the cells were resuspended in D-10 medium. This step was repeated and cells were passed through a 70 μm-cell strainer (BD Bioscience, USA).

**Figure 2:**
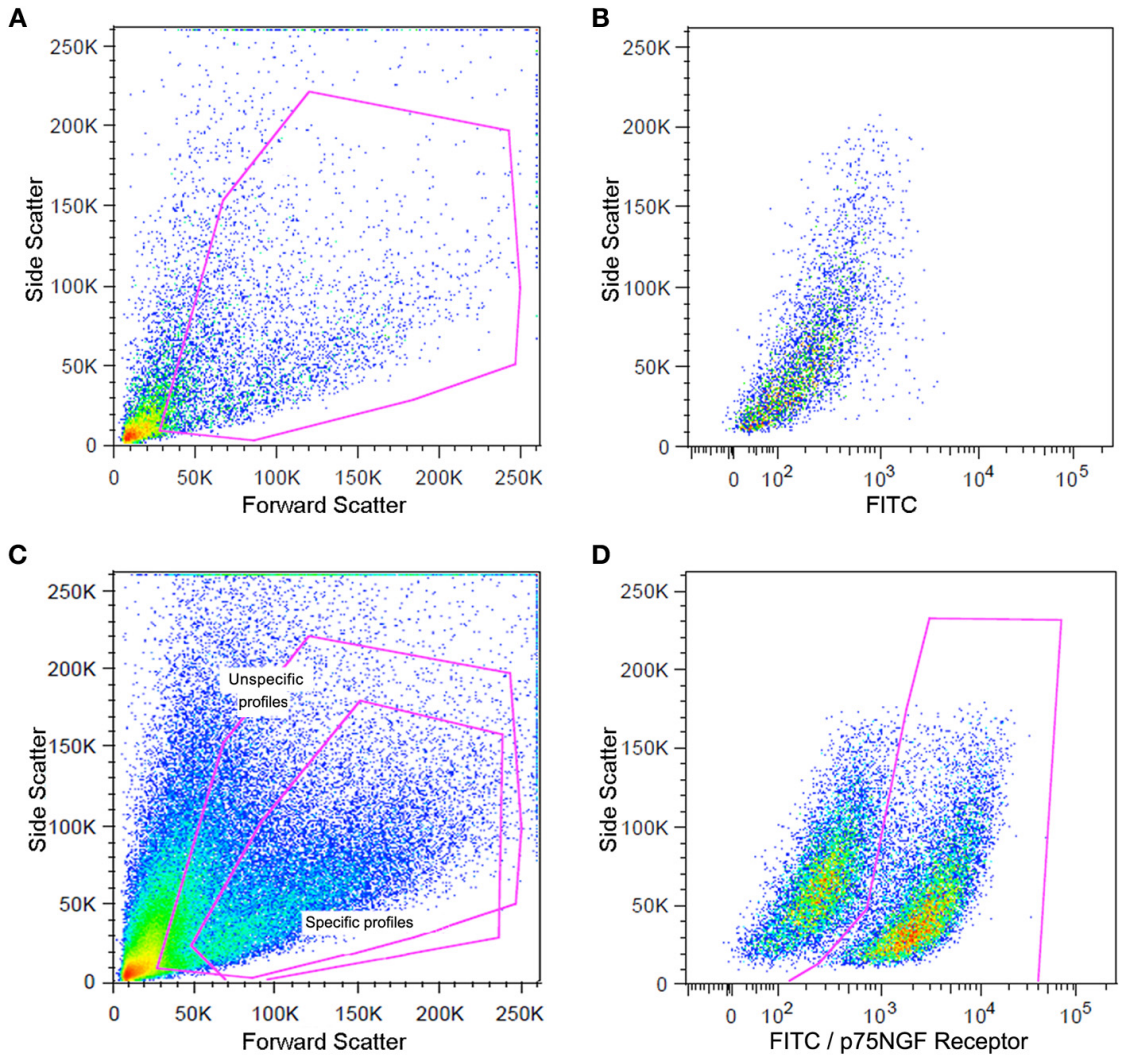
**Flow cytometry analysis of Schwann cells from a mouse sciatic nerve employed in the experiments**. Dot plots indicate the total number of events in the sciatic nerve cell suspension and the dots inside the red box represent the excluded doublet and dead profiles, which have been eliminated by morphological criteria according to previous descriptions (Shapiro, 2005; Herzenberg et al., 2006) **(A)**. Dot plots of unspecific fluorescence are shown in **(B)**. FITIC-conjugated p75NGF receptor antibody was employed in the immunolabeling of Schwann cells **(C,D)**. After morphological criteria, dot plots of labeling profiles **(B)** were identified as unspecific and specific profiles (red boxes) **(C)**. Specific profiles-based on morphological criteria were further analyzed in relation to fluoresce criteria **(B)** and the specific p75NGF receptor positive Schwann cells profiles were identified [red box in **(D)**].

The cells were centrifuged at 1,500 rpm for 5 min at 4°C and the pellets were resuspended in PBS containing 10% fetal bovine serum and 0.01% sodium azide, (Sigma, USA). Sciatic nerve-derived cell suspension was incubated with a fluorescein isothiocyanate (FITC)-conjugated mouse p75NGF receptor antibody (Abcam, USA) diluted in the buffer solution (1/200) for 1 h at room temperature as mentioned above. The p75NGF receptor labeling was employed in the cell sorting experiments because it is a well-characterized surface marker for Schwann cells (Niapour et al., 2010). The samples were then centrifuged (300 × g for 5 min at 4°C). The pellets were washed two times and resuspended in the PBS described above (500 μl). Cells were then analyzed for type and specificity as well as separated on a FACSAria III Cell Sorter (BD Biosciences, USA). A maximum of 10^6^ cells were resuspended in 500 μl of buffer. Flow cytometry dot plot Schwann cell profiles are shown in Figure 2. Details of flow cytometry procedures for cell specificity are described in the supplementary material (Figure S4).

RNA of enriched cells was extracted using Trizol (Life Technologies, USA) according to the manufacturer's protocol. The quantity (NanoDrop 1000 Spectrophotometer) and quality (Agilent 2100 bioanalyser, RNA 6000 Pico LabChip) of RNA were analyzed as described above. Also, the Schwann cell samples were submitted to PCRs in order to access contamination from other cell types. Protocol and results regarding specificity of separated Schwann cell samples are presented in the supplementary material (Figures S3).

### Primary schwann cell culture

**Figure 3:**
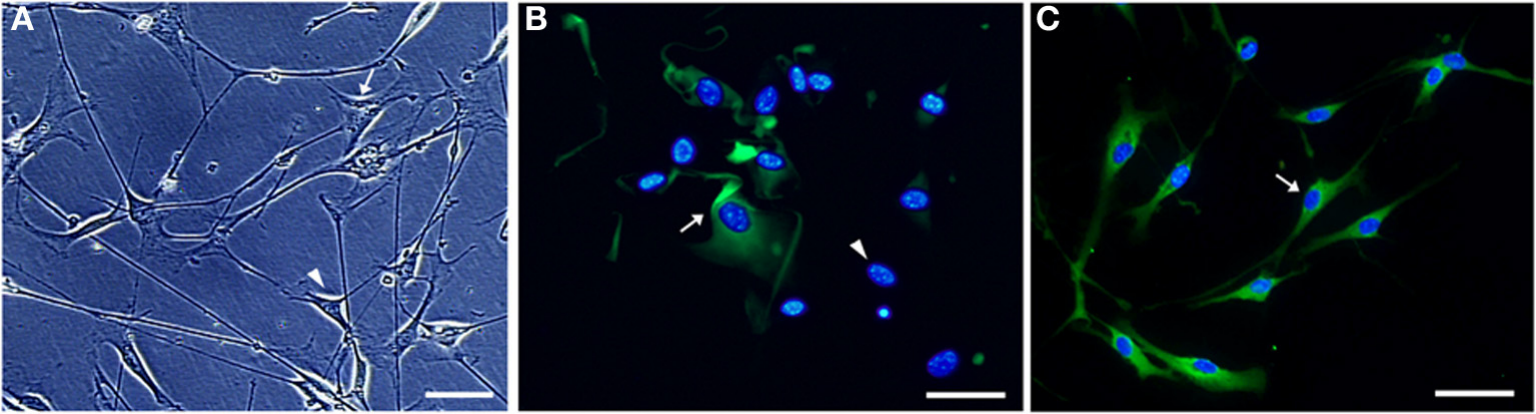
**Microphotographs of Schwann cell cultures obtained from sciatic nerve of a 60 days old SOD1^G93A^ mouse**. Non-purified **(A,B)** and Thy1.2 antibody/rabbit serum complement-eliminated fibroblast [purified, **(C)**] Schwann cell cultures are shown under phase-contrast **(A)** and immunofluorescence **(B,C)** microscopy. Cultured Schwann cells were evidenced by means of S100 immunofluorescence (greenish color), and the cell nuclei (bluish color) were stained by DAPI **(B,C)**. The different morphology of Schwann cells (arrowhead) and fibroblasts (arrow) is observed **(A)**. S100 positive immature Schwann cells (arrow) and DAPI positive nuclei of cells lacking cytoplasmic S100 labeling (arrowhead) are seen in a 24 h plating non-purified culture **(B)**. Vast majority of S100 immunolabeled Schwann cells possess a homogeneous morphology in a 7-days purified culture after fibroblast elimination [arrow, **(C)**]. Scale bars: 50 μm.

Highly purified Schwann cell cultures were obtained from sciatic nerve explants taken from 60 days old SOD1^G93A^ and wild-type mice as described above. Nerve pieces were transferred weekly to new 60 mm dishes filled with 1 ml of D-10 for 5 weeks. Dishes were replaced every other day with a fresh medium (Oudega et al., 1997). After that period, explants were replated onto 35 mm dishes containing a solution of 1.25 U/ml dispase (Boehringer Mannheim, Germany), 0.05% collagenase (Worthington, USA), and D-10, and were kept under overnight incubation in 5% CO2 at 37°C. Following, explants were washed in D-10 and dissociated. The resulting cells were treated with a Thy1.2 antibody (BD Bioscience, USA) and a rabbit serum complement (Calbiochem, USA) for 30 min at room temperature for fibroblast elimination. The protocol for cell enrichment was described elsewhere (Brockes et al., 1979; White et al., 1983; Dong et al., 1999) and was modified according to our experience. The obtained Schwann cells were then seeded onto laminin (Sigma, USA) coated 100 mm dishes for expansion. Twenty-four hours later, the culture medium was replaced by a D-10 medium supplemented with 2 mM forskolin (Sigma, USA) and 20 mg/ml pituitary extract (Gibco, USA). Cells were allowed to expand in that medium until confluence has reached. The medium was changed every other day in the expansion period. The cells of the third passage were used for experiments. Samples of the primary Schwann cell cultures were fixed and immunostained with S100β antibody and nuclei were stained with diamidino-2-phenylindole (DAPI) for cell type verification, as showed in Figure 3.

Schwann cell total RNA was extracted using Trizol (Life Technologies) according to the manufacturer's protocol. The quantity (NanoDrop 1000 Spectrophotometer) and quality (Agilent 2100 bioanalyser, RNA 6000 Pico LabChip) of RNA were analyzed as described above. Cultured Schwann cell RNA samples were submitted to PCRs in order to access fibroblast contamination; the protocol and results are shown in the supplementary material (Figure S3).

### Quantitative PCR

Microarray analyses identified the differentially expressed *Kif1b* in the spinal cord (40 days old mice) and sciatic nerve (60 days old mice) and it was the only gene that its product has been described in the context of ALS (Conforti et al., 2003; Pantelidou et al., 2007). The gene was then selected for verification by qPCR in the whole spinal cord (40 days old mice) and sciatic nerve (60 days) as well as in the enriched motor neurons and Schwann cells. The qPCR verification was performed on independent samples.

Spinal cord cDNA was synthesized from DNAse-treated 1 μg total RNA by employing a TaqMan reverse transcription kit (Applied Biosystems, USA). Sciatic nerve cDNA was synthesized from 100 ng of total RNA by using a Maxima First Strand cDNA Synthesis Kit (Thermo Scientific, USA) according to manufacturers.

qPCR reactions were carried out in duplicate by means of the PikoReal-Time PCR System (Thermo Scientific, USA) employing 40 ng cDNA for spinal cord and 15 ng cDNA for sciatic nerve, the DyNAmo ColorFlash SYBR Green qPCR kit (Thermo Scientific, USA) and finally 400 nM of each primer (***Kif1b***—Forward 5′-3′: CTGCTAGCCCTTTAAGACTCG; Reverse 5′-3′: AAACTCCTAGACAAACGCTCC; ***Gapdh***—Forward 5′-3′: GAGTAAGAAACCCTGGACCAC; Reverse 5′-3′: TCTGGGATGGAAATTGTGAGG) in a 20 μl final volume reaction.

The *Kif1b* expression was also evaluated in the enriched microdissected Schwann cells and motor neurons as well as in the Schwann cells enriched by means of cell sorting and primary culture procedures. cDNA samples of microdissected cells and of cultured Schwann cells were synthesized from 1 μg of amplified RNA as described previously (De Oliveira et al., 2013). The cDNA of flow cytometry sorting Schwann cells was synthesized from 100 ng of total RNA.

The cycling for SYBR reactions was composed by an initial denaturation at 95°C for 10 min. Templates were amplified by 40 cycles of 95°C for 15 s and of 60°C for 30 s. A dissociation curve was then generated to ensure amplification of a single product and absence of primer dimers. A standard curve was generated for each primer pair in order to determine the efficiency of the PCR reaction over a range of template concentrations from 0.032 to 20 ng/μ l, using cDNA synthesized from reference mouse RNA. The efficiency for each set of primers was 100 ± 5%. Gene expressions, which were normalized by *Gapdh*, could be determined using the ΔΔCt mathematical model (ABI PRISM 7700 Sequence Detection System protocol; Applied Biosystems). *Gapdh* was chosen as a housekeeping gene to normalize the qPCR values because the microarray analysis showed no alteration in the gene expression across samples.

### Statistical analysis

The statistical method employed in the microarray analysis is described above (Hulsen et al., 2008). Furthermore, one-tailed unpaired t-test was used to determine the statistical significance of differences in gene expression [Graphpad Prism 5 (San Diego, CA)] in the qPCR analyses.

## Results

### Microarray analysis

The DAVID analysis of differentially expressed genes of 40 days old SOD1^G93A^ mice pointed 34 enriched GO terms under high stringency conditions. The CCO indicated only one GO term related to cytoskeleton, the actin cytoskeleton, with 14 genes (six down and eight upregulated), which are shown in Table 1. DAVID also pointed 63 enriched terms from the differentially expressed genes of spinal cord of 80 days old SOD1^G93A^ mice (Table 2). The CCO indicated five GO terms related to cytoskeleton (Table 2) in the spinal cord of 80 days old SOD1^G93A^ mice, specifically the microtubule cytoskeleton (35 genes), cytoskeleton part (53 genes), actin cytoskeleton (16 genes), neurofilament cytoskeleton (three genes), and cytoskeleton (76 genes). Those genes are overlapped with the 76 deregulated genes of cytoskeleton category (25 down and 51 upregulated).

**Table 1:** **List of differentially expressed genes in spinal cord of 40 days old SOD1^G93A^ mice related to cytoskeleton**.

**Probe set ID**	**Gene symbol**	**Gene name**	**Fold change**
A_52_P654108	*Dync1li2*	dynein, cytoplasmic 1, light intermediate chain 2	1.11
A_51_P319551	*Kif3a*	kinesin family member 3A	−1.13
A_51_P264956	*Kif1b*	kinesin family member 1B	1.09
A_51_P438349	*Kif1c*	kinesin family member 1C	1.14
A_52_P581390	*Kif1c*	kinesin family member 1C	1.08
A_52_P56751	*Lcp1*	lymphocyte cytosolic protein 1	−1.14
A_51_P316103	*Lima1*	LIM domain and actin binding 1	−1.16
A_52_P274238	*Maea*	macrophage erythroblast attacher	1.10
A_51_P362429	*Myh11*	myosin, heavy polypeptide 11, smooth muscle	−1.12
A_52_P241519	*Myo1c*	similar to nuclear myosin I beta; myosin IC	−1.15
A_51_P185141	*Myo1e*	myosin IE	1.08
A_51_P440923	*Sh3pxd2a*	SH3 and PX domains 2A; similar to Fish	−1.19
A_52_P155100	*Srcin1*	P140 gene	1.29
A_51_P436534	*Twf1*	twinfilin, actin-binding protein, homolog 1 (Drosophila)	1.10
A_52_P96782	*Wasl*	Wiskott-Aldrich syndrome-like (human)	1.16

The Kif1c has been demonstrated in the list by two different Probes Set IDs.

**Table 2:** **List of differentially expressed genes in spinal cord of 80 days old SOD1^G93A^ mice related to cytoskeleton**.

**Probe set ID**	**Gene symbol**	**Gene name**	**Fold change**
A_52_P485007	*Abca2*	ATP-binding cassette, sub-family A (ABC1), member 2	1.23
A_52_P209101	*Abl1*	c-abl oncogene 1, receptor tyrosine kinase	1.09
A_52_P72237	*Actg1*	predicted gene 8543; actin-like 8; predicted gene 7505	1.34
A_51_P188845	*Adora1*	adenosine A1 receptor	1.15
A_52_P418014	*Akt1*	thymoma viral proto-oncogene 1	−1.09
A_51_P269375	*Ank1*	ankyrin 1, erythroid; hypothetical protein LOC100046690	1.13
A_51_P319562	*Ank2*	ankyrin 2, brain	−1.26
A_51_P318104	*App*	amyloid beta (A4) precursor protein	−1.16
A_52_P58041	*Arpc5*	predicted gene 16372; actin related protein 2/3 complex, subunit 5	1.18
A_52_P1157979	*Calm3*	predicted gene 7743; calmodulin 3; calmodulin 2; calmodulin 1	1.45
A_51_P440682	*Cap1*	CAP, adenylate cyclase-associated protein 1 (yeast)	−1.39
A_51_P135423	*Capzb*	capping protein (actin filament) muscle Z-line, beta	1.15
A_51_P180629	*Cdc42ep1*	CDC42 effector protein (Rho GTPase binding) 1	1.14
A_51_P128148	*Chmp1a*	chromatin modifying protein 1A; predicted gene 8515	1.34
A_52_P479539	*Cit*	citron	1.37
A_51_P420547	*Clic5*	chloride intracellular channel 5	−1.13
A_52_P326214	*Cttn*	cortactin; predicted gene 8786	1.25
A_51_P483908	*Dctn1*	dynactin 1	1.26
A_51_P219868	*Dnm1*	dynamin 1	1.16
A_51_P448458	*Dnm3*	dynamin 3	−1.25
A_52_P184304	*Dst*	dystonin; hypothetical protein LOC100047109	1.15
A_52_P429909	*Dynll2*	dynein light chain LC8-type 2	1.23
A_51_P227962	*Dynlrb2*	dynein light chain roadblock-type 2	−1.09
A_52_P371946	*Eif6*	eukaryotic translation initiation factor 6	1.14
A_51_P184806	*Elmod2*	ELMO domain containing 2	−1.12
A_51_P360622	*Elmod3*	ELMO/CED-12 domain containing 3	1.10
A_52_P396917	*Eml5*	echinoderm microtubule associated protein like 5	−1.12
A_52_P524426	*Epb4.1l1*	erythrocyte protein band 4.1-like 1	1.15
A_52_P621940	*Epb4.1l2*	erythrocyte protein band 4.1-like 2	1.16
A_52_P684050	*Fam110a*	family with sequence similarity 110, member A	−1.23
A_52_P27871	*Fnbp1*	formin binding protein 1	1.20
A_51_P304757	*Gabarapl1*	gamma-aminobutyric acid (GABA) A receptor-associated protein-like 1	1.49
A_51_P241465	*Gsn*	gelsolin	1.24
A_52_P212597	*Hook1*	hook homolog 1 (Drosophila)	−1.15
A_52_P247513	*Hook3*	hook homolog 3 (Drosophila)	−1.17
A_52_P49378	*Kif1a*	kinesin family member 1A	1.24
A_52_P282500	*Kif21b*	kinesin family member 21B	1.20
A_51_P193011	*Klc1*	kinesin light chain 1	1.26
A_51_P363396	*Klc2*	kinesin light chain 2	1.24
A_51_P259118	*Klhl1*	kelch-like 1 (Drosophila)	−1.32
A_51_P312348	*Krt7*	keratin 7	−1.14
A_51_P242399	*Krt8*	keratin 8	−1.13
A_52_P419298	*Lasp1*	LIM and SH3 protein 1	1.19
A_51_P386638	*Llgl1*	lethal giant larvae homolog 1 (Drosophila)	1.19
A_51_P411645	*Maea*	macrophage erythroblast attacher	1.19
A_51_P126177	*Map1lc3b*	microtubule-associated protein 1 light chain 3 beta	1.16
A_52_P327537	*Mpdz*	multiple PDZ domain protein	1.16
A_51_P318580	*Myh14*	myosin, heavy polypeptide 14	1.18
A_51_P512210	*Myh6*	myosin, heavy polypeptide 6, cardiac muscle, alpha	−1.16
A_52_P544523	*Myl4*	myosin, light polypeptide 4	−1.13
A_52_P650855	*Myo1d*	myosin ID	1.22
A_51_P114062	*Ncs1*	frequenin homolog (Drosophila)	1.14
A_51_P145220	*Nefm*	neurofilament, medium polypeptide	1.30
A_51_P238933	*Nudc*	nuclear distribution gene C homolog (Aspergillus)	−1.13
A_52_P89425	*Pcnt*	pericentrin (kendrin)	1.13
A_51_P472726	*Pdlim2*	PDZ and LIM domain 2	1.31
A_51_P270478	*Pin4*	protein (peptidyl-prolyl cis/trans isomerase) NIMA-interacting, 4 (parvulin)	−1.16
A_52_P359381	*Ptk2*	PTK2 protein tyrosine kinase 2	1.12
A_51_P275679	*Rassf5*	Ras association (RalGDS/AF-6) domain family member 5	1.14
A_52_P24320	*Rpgrip1l*	Rpgrip1-like	−1.13
A_52_P656024	*Sirt2*	sirtuin 2 (silent mating type information regulation 2, homolog)	1.19
A_51_P371311	*Slc1a4*	solute carrier family 1 (glutamate/neutral amino acid transporter), member 4	1.13
A_51_P495641	*Stmn1*	stathmin 1; predicted gene 11223; predicted gene 6393	1.14
A_51_P264634	*Strbp*	spermatid perinuclear RNA binding protein	1.15
A_51_P404875	*Synm*	synemin, intermediate filament protein	1.15
A_52_P261322	*Tanc1*	tetratricopeptide repeat, ankyrin repeat, and coiled-coil containing 1	1.13
A_51_P224843	*Tmsb4x*	thymosin, beta 4, X chromosome; similar to thymosin beta-4	−1.21
A_51_P507899	*Ttc8*	tetratricopeptide repeat domain 8	−1.10
A_51_P169745	*Tuba1a*	predicted gene 7172; similar to tubulin, alpha 1; tubulin, alpha 1A	1.25
A_52_P490023	*Tubb2a*	tubulin, beta 2A	1.19
A_52_P621603	*Tubb2a*	tubulin, beta 2A	1.26
A_52_P97417	*Tubgcp5*	tubulin, gamma complex associated protein 5	−1.10
A_52_P266540	*Ubr4*	ubiquitin protein ligase E3 component n-recognin 4	1.17
A_52_P569218	*Utrn*	utrophin	−1.10
A_51_P361788	*Vapa*	vesicle-associated membrane protein, associated protein A	−1.10
A_52_P219314	*Vasp*	vasodilator-stimulated phosphoprotein	1.08
A_51_P473252	*Zyx*	zyxin	1.15

The Tubb2a has been demonstrated in the list by two different Probes Set IDs.

The DAVID analysis also pointed 55 enriched terms from the deregulated genes of sciatic nerve of 60 days old SOD1^G93A^ mice. Furthermore, the CCO indicated four GO terms related with cytoskeleton, specifically the actin cytoskeleton (43 genes), cytoskeleton part (101 genes), microtubule cytoskeleton (64 genes), and cytoskeleton (146 genes). The 146 genes of the cytoskeleton GO term (74 down and 72 upregulated) are overlapped with all other GO terms (Table 3).

**Table 3:** **List of differentially expressed genes in sciatic nerve of 60 days old SOD1^G93A^ mice related to cytoskeleton**.

**Probe set ID**	**Gene symbol**	**Gene name**	**Fold change**
A_55_P2024808	*Abl1*	c-abl oncogene 1, receptor tyrosine kinase	1.19
A_52_P489778	*Ablim1*	actin-binding LIM protein 1	1.30
A_51_P246854	*Acta1*	actin, alpha 1, skeletal muscle	4.08
A_52_P420504	*Acta2*	actin, alpha 2, smooth muscle, aorta	2.08
A_55_P1963807	*Actg2*	actin, gamma 2, smooth muscle, enteric	2.45
A_52_P656699	*Actn3*	actinin alpha 3	2.81
A_51_P400543	*Aif1*	allograft inflammatory factor 1	−1.38
A_52_P311297	*Als2*	amyotrophic lateral sclerosis 2 (juvenile) homolog (human)	1.21
A_55_P1979156	*Arap3*	ArfGAP with RhoGAP domain, ankyrin repeat, and PH domain 3	1.21
A_52_P195018	*Arap3*	ArfGAP with RhoGAP domain, ankyrin repeat, and PH domain 3	1.32
A_55_P2047986	*Ankrd23*	ankyrin repeat domain 23	1.42
A_55_P2021810	*Arc*	activity regulated cytoskeletal-associated protein	−1.44
A_52_P153189	*Arl2bp*	ADP-ribosylation factor-like 2 binding protein	−1.16
A_55_P2023076	*Arpc1b*	actin related protein 2/3 complex, subunit 1B; Arpc1b	−1.63
A_52_P369581	*Atm*	ataxia telangiectasia mutated homolog (human)	1.17
A_52_P400509	*Atm*	ataxia telangiectasia mutated homolog (human)	1.18
A_55_P1980636	*Aurka*	aurora kinase A; Aurka	−1.27
A_55_P1983768	*Birc5*	baculoviral IAP repeat-containing 5	−1.23
A_55_P2029106	*Bmf*	BCL2 modifying factor	1.28
A_51_P357573	*Cald1*	caldesmon 1	1.18
A_52_P140356	*Calm3*	calmodulin 3	−1.29
A_66_P106654	*Camsap1*	calmodulin regulated spectrin-associated protein 1	−1.26
A_55_P2065671	*Ccnb1*	cyclin B1	−1.60
A_52_P155554	*Cdc42ep2*	CDC42 effector protein (Rho GTPase binding) 2	−1.24
A_51_P267494	*Cdc42ep3*	CDC42 effector protein (Rho GTPase binding) 3	1.54
A_55_P2043269	*Cdc42se1*	CDC42 small effector 1	−1.20
A_51_P155142	*Cdca8*	cell division cycle associated 8	−1.17
A_52_P162099	*Ckap2*	cytoskeleton associated protein 2	−1.22
A_51_P420547	*Clic5*	chloride intracellular channel 5	1.32
A_51_P351194	*Cnfn*	cornifelin	1.21
A_51_P109258	*Cys1*	cystin 1	1.20
A_51_P357085	*Dctn6*	dynactin 6	1.27
A_51_P335969	*Des*	desmin	1.38
A_55_P2050439	*Dlgap5*	discs, large (Drosophila) homolog-associated protein 5	−1.42
A_55_P2119907	*Dnahc11*	dynein, axonemal, heavy chain 11	−1.23
A_52_P485891	*Dnahc5*	dynein, axonemal, heavy chain 5	−1.20
A_51_P459350	*Dstn*	destrin	1.32
A_55_P2090429	*Dync1i1*	dynein cytoplasmic 1 intermediate chain 1	−1.43
A_52_P654108	*Dync1li2*	dynein, cytoplasmic 1 light intermediate chain 2	−1.20
A_51_P203878	*Dynll2*	dynein light chain LC8-type 2	−1.17
A_51_P203878	*Dynll2*	dynein light chain LC8-type 2	−1.23
A_55_P2069949	*Dynlrb1*	dynein light chain roadblock-type 1	−1.22
A_55_P2113673	*Eml1*	echinoderm microtubule associated protein like 1	−1.23
A_55_P1960097	*Epb4.1l3*	erythrocyte protein band 4.1-like 3	−1.15
A_55_P1956488	*Epb4.9*	erythrocyte protein band 4.9	1.26
A_66_P110161	*Eppk1*	epiplakin 1	1.34
A_51_P440865	*Fam110b*	family with sequence similarity 110, member B	1.19
A_51_P512783	*Fam82b*	family with sequence similarity 82, member B	1.30
A_52_P330395	*Farp1*	FERM, RhoGEF (Arhgef), and pleckstrin domain protein 1	1.19
A_55_P2029051	*Fgd3*	FYVE, RhoGEF, and PH domain containing 3	−1.48
A_52_P493620	*Fgfr1op*	Fgfr1 oncogene partner	−1.30
A_51_P495379	*Fhod1*	formin homology 2 domain containing 3	1.31
A_55_P2088018	*Fhod3*	formin homology 2 domain containing 3	−1.27
A_51_P495379	*Flna*	filamin, alpha	1.31
A_55_P2425801	*Fmn1*	formin 1	1.18
A_55_P2057537	*Gas7*	growth arrest specific 7	−1.17
A_55_P2025403	*Gphn*	gephyrin	1.14
A_51_P506748	*Grlf1*	glucocorticoid receptor DNA binding factor 1	−1.26
A_51_P214306	*Haus4*	HAUS augmin-like complex, subunit 4	1.17
A_51_P440460	*Hip1r*	huntingtin interacting protein 1 related	1.15
A_51_P346445	*Hspb7*	heat shock protein family, member 7 (cardiovascular)	1.36
A_51_P391445	*Ifngr1*	interferon gamma receptor 1	1.14
A_55_P1978201	*Incenp*	inner centromere protein	−1.29
A_55_P2178044	*Inppl1*	inositol polyphosphate phosphatase-like 1	−1.34
A_51_P218653	*Jph2*	junctophilin 2	1.26
A_55_P2008066	*Itpr1*	inositol 1,4,5-triphosphate receptor 1	1.27
A_51_P400016	*Kalrn*	kalirin, RhoGEF kinase	1.48
A_51_P493857	*Katna1*	katanin p60 (ATPase-containing) subunit A1	−1.14
A_55_P2184741	*Katnal1*	katanin p60 subunit A-like 1	−1.29
A_65_P12993	*Kif1b*	kinesin family member 1B	−1.40
A_52_P581390	*Kif1c*	kinesin family member 1C	1.35
A_51_P133137	*Kif20a*	kinesin family member 20A	−1.27
A_51_P324287	*Kif23*	kinesin family member 23	−1.23
A_51_P254805	*Kif4*	kinesin family member 4	−1.19
A_51_P107020	*Kif5a*	kinesin family member 5A	1.25
A_66_P116311	*Kif5b*	kinesin family member 5B	−1.20
A_55_P2048937	*Kif5c*	kinesin family member 5C	1.42
A_51_P154753	*Klc3*	kinesin light chain 3	−1.37
A_52_P410685	*Krt7*	keratin 7	1.16
A_55_P2086334	*Krt85*	keratin 85	1.25
A_52_P642801	*Lats1*	large tumor suppressor	−1.23
A_55_P2066613	*Lcp1*	lymphocyte cytosolic protein 1	−1.18
A_65_P01834	*Lima1*	LIM domain and actin binding 1	1.18
A_51_P120717	*Lmnb1*	lamin B1	−1.21
A_55_P2017684	*Macf1*	microtubule-actin crosslinking factor 1	1.19
A_55_P2009091	*Mad1l1*	MAD1 mitotic arrest deficient 1-like 1	−1.18
A_55_P2142151	*Mapk1ip1*	mitogen-activated protein kinase 1 interacting protein 1	1.23
A_55_P1954486	*Mapt*	microtubule-associated protein tau	−1.17
A_55_P2004777	*Micall2*	MICAL-like 2	−1.42
A_51_P124568	*Mpp1*	membrane protein, palmitoylated	1.54
A_55_P2147280	*Myh1*	myosin, heavy polypeptide 1, skeletal muscle, adult	2.80
A_55_P1988531	*Myh11*	myosin, heavy polypeptide 11, smooth muscle	2.35
A_51_P416858	*Myl1*	myosin, light polypeptide 1	5.53
A_66_P107790	*Myl12a*	myosin, light chain 12A	1.44
A_55_P2107045	*Myl4*	myosin, light polypeptide 4	1.20
A_51_P308298	*Myl9*	myosin, light polypeptide 9, regulatory	1.33
A_51_P324303	*Mylip*	myosin regulatory light chain interacting protein	−1.18
A_55_P2154049	*Myo18a*	myosin XVIIIA	−1.18
A_55_P1955034	*Myo1c*	similar to nuclear myosin I beta; myosin IC	1.20
A_52_P650855	*Myo1d*	myosin ID	1.27
A_55_P2006250	*Myo5a*	myosin VA	−1.19
A_66_P115949	*Myo9a*	myosin Ixa	−1.15
A_51_P114062	*Ncs1*	neuronal calcium sensor 1	−1.19
A_55_P2116978	*Neb*	nebulin	1.52
A_52_P367520	*Nexn*	nexilin	1.16
A_55_P2423646	*Nf2*	neurofibromatosis 2	1.21
A_55_P2155582	*Nin*	ninein	−1.24
A_55_P2158741	*Nos2*	nitric oxide synthase 2, inducible	−1.26
A_51_P139651	*Nos3*	nitric oxide synthase 3, endothelial cel	1.55
A_51_P240453	*Nusap1*	nucleolar and spindle associated protein 1	−1.23
A_55_P2058137	*Pde4dip*	phosphodiesterase 4D interacting protein (myomegalin)	2.21
A_51_P472726	*Pdlim2*	PDZ and LIM domain 2	1.43
A_52_P579531	*Pdlim3*	PDZ and LIM domain 3	2.07
A_55_P2004571	*Pitpnm2*	phosphatidylinositol transfer protein, membrane-associated 2	1.17
A_52_P234729	*Pkd2*	polycystic kidney disease 2	−1.27
A_52_P668285	*Plk4*	polo-like kinase 4	−1.19
A_55_P1988083	*Prc1*	protein regulator of cytokinesis 1	−1.38
A_51_P382152	*Procr*	protein C receptor, endothelial	1.50
A_55_P2429225	*Psrc1*	proline/serine-rich coiled-coil 1	−1.18
A_51_P455946	*Rac3*	RAS-related C3 botulinum substrate 3	−1.19
A_55_P2127702	*Racgap1*	Rac GTPase-activating protein 1	−1.20
A_51_P221337	*Ranbp10*	RAN binding protein 10	1.16
A_52_P76034	*Rcc2*	regulator of chromosome condensation 2	−1.18
A_51_P227392	*Rhou*	ras homolog gene family, member U	−1.32
A_51_P435922	*Rsph9*	radial spoke head 9 homolog (Chlamydomonas)	−1.44
A_55_P2168628	*Sac3d1*	SAC3 domain containing 1	−1.14
A_51_P389004	*Sgcd*	sarcoglycan, delta (dystrophin-associated glycoprotein)	1.17
A_51_P115626	*Shank3*	SH3/ankyrin domain gene 3	1.22
A_52_P78373	*Sorbs3*	sorbin and SH3 domain containing 3	1.31
A_51_P513530	*Spag5*	sperm associated antigen 5	−1.28
A_51_P348652	*Spast*	spastin	−1.34
A_51_P386870	*Sprr2f*	small proline-rich protein 2F	−1.19
A_55_P2081123	*Srcin1*	SRC kinase signaling inhibitor 1	−0.30
A_55_P1988043	*Ssh1*	slingshot homolog 1 (Drosophila)	−1.15
A_55_P1968977	*Stk38l*	serine/threonine kinase 38 like	−1.19
A_52_P639064	*Strbp*	spermatid perinuclear RNA binding protein	−1.23
A_51_P123676	*Synpo*	synaptopodin	1.19
A_55_P2004801	*Tacc3*	transforming, acidic coiled-coil containing protein 3	−1.18
A_51_P429276	*Tmod3*	tropomodulin 3	1.36
A_55_P2008895	*Tmsb15b1*	thymosin beta 15b1	1.41
A_52_P315976	*Tpm2*	tropomyosin 2, beta	2.00
A_55_P2121408	*Tpm2*	tropomyosin 2, beta	2.29
A_51_P369200	*Tpx2*	TPX2, microtubule-associated protein homolog (Xenopus laevis)	−1.20
A_51_P208697	*Ttl*	tubulin tyrosine ligase	−1.43
A_66_P119518	*Tuba8*	tubulin, alpha 8	−1.24
A_51_P514256	*Tubb2b*	tubulin, beta 2B class IIB	−1.34
A_55_P2034864	*Tubb2b*	tubulin, beta 2B class IIB	−1.32
A_55_P2013645	*Tubg2*	tubulin, gamma 2	1.17
A_51_P226932	*Tubgcp2*	tubulin, gamma complex associated protein 2	1.18
A_52_P484405	*Twf1*	twinfilin, actin-binding protein, homolog 1 (Drosophila)	−1.15
A_52_P190973	*Vcl*	vinculin	1.25
A_55_P1963443	*Vps18*	vacuolar protein sorting 18 (yeast)	1.17

Each Arap3, Atm, Dynll2, Tpm2, and Tubb2b have been demonstrated in the list by two different Probes Set IDs.

**Figure 4:**
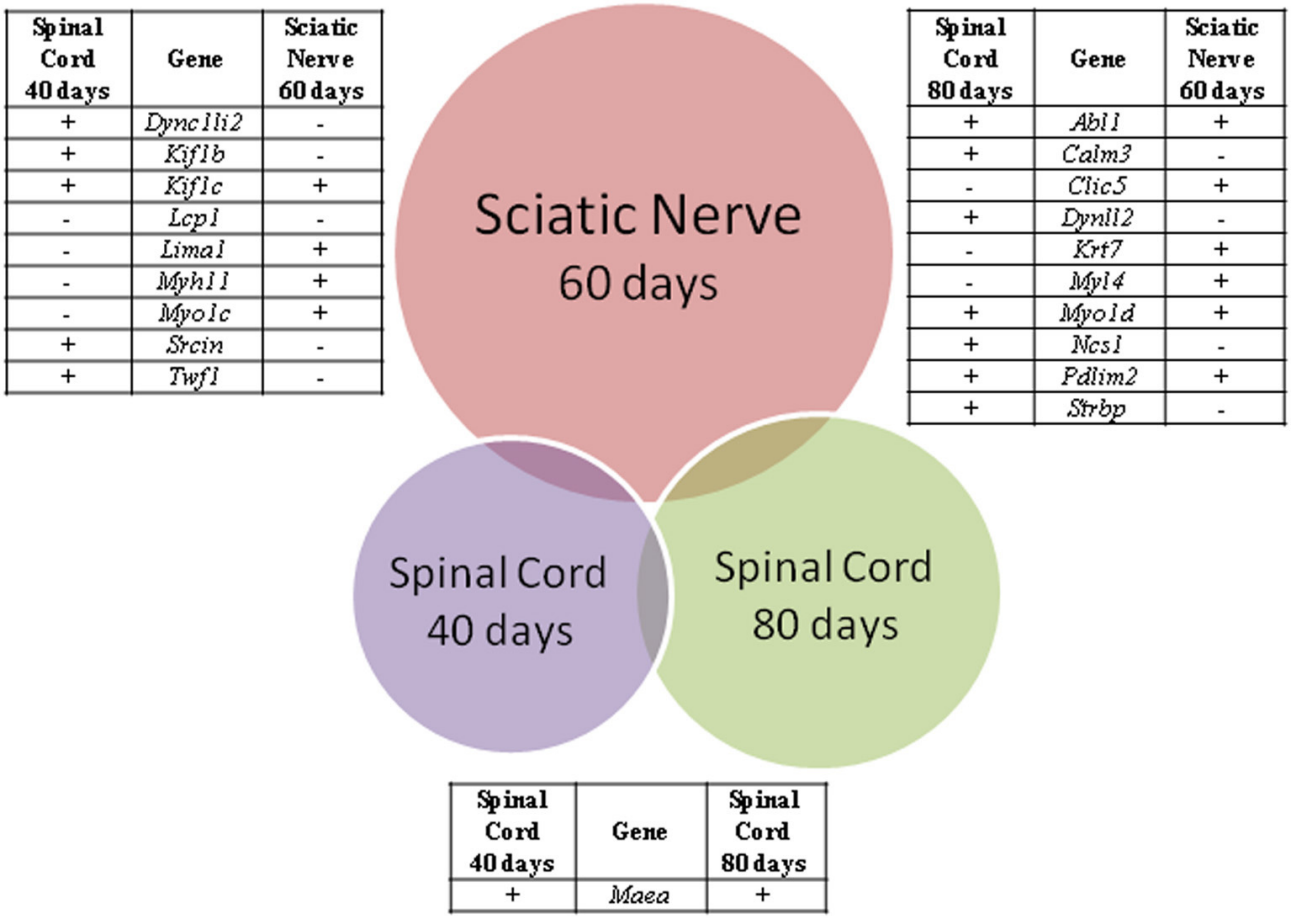
**Venn diagram of differentially expressed genes related to cytoskeleton analysis in spinal cords (40 and 80 days) and sciatic nerve (60 days) of SOD1^G93A^ animals compared to wild-type controls by means of microarray experiments**. The enrichment cytoskeleton lists were obtained by means of DAVID tool based on Cellular Component Ontology, which identified 146 differentially expressed genes in the sciatic nerve from 60 days old mice, 76 genes in spinal cord from 80 days old mice and 14 genes in spinal cord from 40 days old mice. Venn diagram demonstrated nine genes common between sciatic nerve and 40 days old mouse spinal cord, 10 genes common between sciatic nerve and 80 days old mouse spinal cord, and only one gene common between spinal cord groups. Positive (+) and negative (−) signals represent the upregulated and the down regulated genes, respectively.

From the above lists of differentially expressed genes related to cytoskeleton pointed by CCO, nine genes (*Dync1li2*, *Kif1b*, *Kif1c*, *Lcp1*, *Lima1*, *Myh11*, *Myo1c*, *Srcin*, *Twf1*) were deregulated in both spinal cord and sciatic nerve of SOD1^G93A^ mice at the presymptomatic ages 40 and 60 days, respectively, and 10 genes (*Abl1*, *Calm3*, *Clic5*, *Dynll2*, *Krt7*, *Myl4*, *Myo1d*, *Ncs1*, *Pdlim2*, *Strbp*) were deregulated in the above regions of 80 and 60 days mice, respectively, as shown by the Venn diagram (Figure 4). Furthermore, only the *Maea* was seen differentially expressed in the spinal cord of 40 and 80 days old presymptomatic SOD1^G93A^ mice and no gene appeared to repeat in the three lists of the studied regions of the presymptomatic mice (Figure 4). It should be pointed out that only the *Kif1b* from the above described differentially expressed genes related to cytoskeleton has been already mentioned in the context of ALS and has been detailed in the present cellular and molecular analyses.

### Motor neuron and schwann cell enrichment

In order to analyze the modulation of gene expression in specific cell populations possibly involved in the pathogenic mechanisms in ALS, spinal cord motor neurons (lumbar regions) were obtained by means of single cell laser microdissection and the sciatic nerve Schwann cells were achieved by means of laser microdissection, flow cytometry cell sorting and cell culture. The levels of cDNA specific cell type marker that demonstrated the enrichment for each cell type obtained by respective technique are shown in the Supplementary Material (S2, S3).

### *Kif1b* regulation evidenced by qPCR

**Figure 5:**
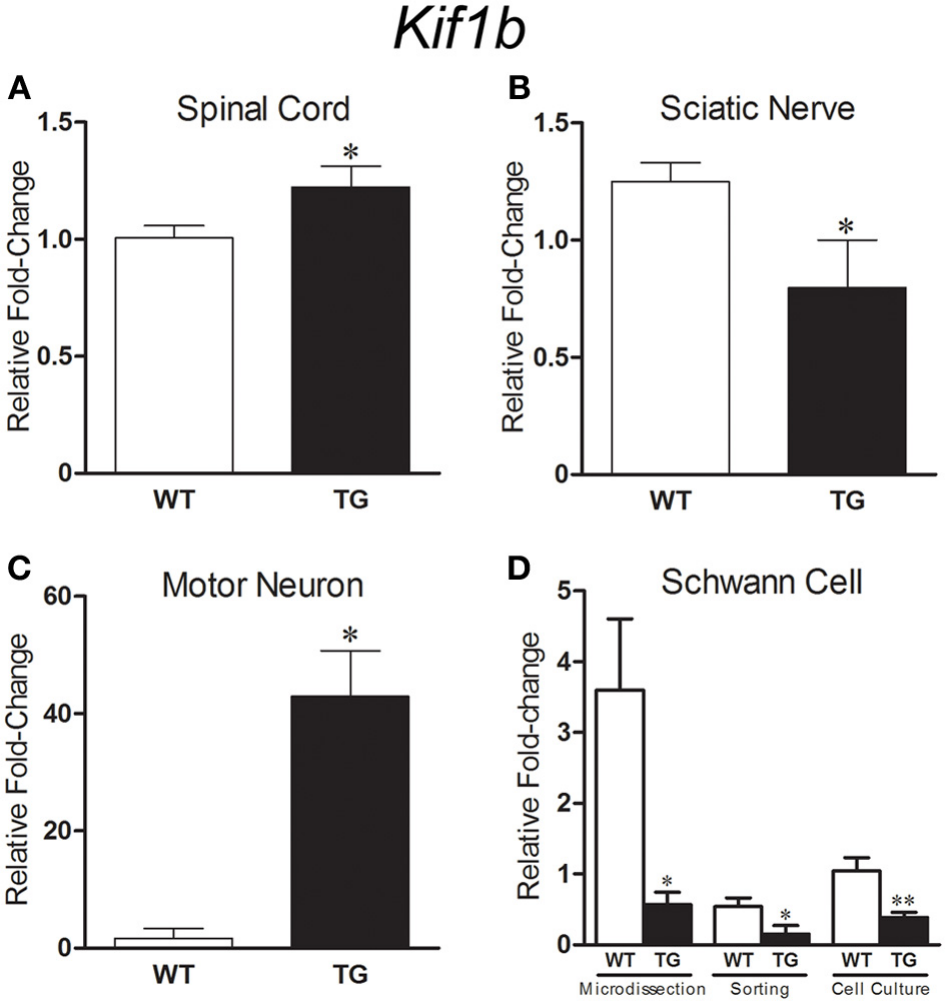
**Graphs show relative fold change values for *Kif1b* in SOD1^G93A^ (TG) and age matched wild-type controls (WT) mice by means of qPCR**. Gene expression differences in the spinal cord [40 days old, **(A)**], sciatic nerve [60 days old, **(B)**] and microdissected motor neurons [40 days old, **(C)**] are pointed. *Kif1b* expression was downregulated in sciatic nerve Schwann cell [60 days old, **(D)**] enriched samples from microdissection, flow cytometry sorting and cell culture procedures. Means ± SEM from four samples of each group. ^*^ and ^**^ *p*-values, < 0.05 and < 0.01, respectively, according to unpaired *t* test.

qPCR analyses of *Kif1b* expression showed an upregulation (1.21 fold) of the gene in spinal cord of presymptomatic 40 days old SOD1^G93A^ mice (Figure 5A) and a downregulation (1.57 fold) in the sciatic nerve of presymptomatic 60 days old transgene mice (Figure 5B). These regulations were coincident and supported the microarray findings. Additionally, qPCR analyses also demonstrated the differentially expression of *Kif1b* in enriched cell assays using two cycle amplified RNA. Upregulation of *Kif1b* (24.8 fold) was seen in laser microdissected motor neurons from 40 days old SOD1^G93A^ mice (Figure 5C); this regulation was in the same direction to that found in the whole spinal cord preparation of 40 days transgene mice by means of microarray and qPCR analyses. Remarkably, *Kif1b* was downregulated in the enriched sciatic nerve Schwann cells (60 days old SOD1^G93A^ mice) by means of single cell laser microdissection (6.35 fold), cell sorting (3.53 fold), and cell culture (2.70 fold) (Figure 5D), regulations that were in the same direction to that found in the whole sciatic nerve preparation of 60 days transgene mice by means of microarray and qPCR analyses.

## Discussion

### Cytoskeleton enrichment analysis of gene profiling in presymptomatic ALS

Gene regulation of cytoskeleton-related molecules employing microarray technology has been described in the spinal cord and/or microdissected survival neurons from post mortem material of ALS patients (Jiang et al., 2005; Offen et al., 2009; Cox et al., 2010; Tanaka et al., 2012), thus reflecting the cytoskeleton responses to injury instead its role on neurodegenerative triggering. Sporadic and unsystematic results on deregulated genes-related to cytoskeleton in ALS animal models have been demonstrated in several stages of the disease (Perrin et al., 2005; Tanaka et al., 2006; Ferraiuolo et al., 2007; Guipponi et al., 2010; Kudo et al., 2010). The present study extends previous descriptions by detailing gene profiling in expanded categories of cytoskeleton-related genes in the presymptomatic ages of the ALS mouse model. The DAVID analysis was applied by using the full lists of deregulated genes of the spinal cord of 40 and 80 days old presymptomatic ALS mice we have published recently (De Oliveira et al., 2013). Our study has also demonstrated for the first time the gene profiling of cytoskeleton category in the peripheral nerve (sciatic nerve) of the ALS model in a presymptomatic period of the disease.

It should be highlighted the large number of deregulated genes of the cytoskeleton-related category in the spinal cord of presymptomatic ALS mouse of the present study compared to previous descriptions that employed different methodologies (Ferraiuolo et al., 2007; Guipponi et al., 2010; Kudo et al., 2010). In fact, from 13 deregulated genes grouped in categories of cytoskeleton and transport described in the presymptomatic spinal cord of a late onset ALS animal model (Guipponi et al., 2010), the down regulation of kinesin light chain 2 (*Klc2*) gene was in agreement to our analysis. Also, from 11 deregulated genes of the cytoskeleton and motor activity categories described in microdissected toluidine blue-labeled neurons from the spinal cord of asymptomatic SOD1^G93A^ mice (Ferraiuolo et al., 2007), the upregulated *Kif1b* and *Gsn* were found, respectively, in spinal cord of 40 and 80 days old mice of our analysis. Furthermore, similar study that employed a categorization of enriched pathways in asymptomatic ALS mice has not identified cytoskeleton category but pointed six differentially expressed cytoskeleton genes (Kudo et al., 2010).

It is interesting the robust regulation of gene expression in the spinal cord of the ALS mouse model in the presymptomatic phases of the disease, thus underlining the early events on modulation of cytoskeleton elements before the death of spinal cord motor neurons. The genes of cellular components related to cytoskeleton pointed by DAVID enriched analysis showed 14 and 76 deregulated genes in the spinal cord of 40 and 80 days old presymptomatic animals respectively, and 146 deregulated genes in the sciatic nerve of 60 days presymptomatic ALS mice.

In fact, early events regarding impairment of axonal transport, cytoplasm aggregation and neurite/axonal abnormalities have been described before the onset of ALS symptoms (Warita et al., 1999; Williamson and Cleveland, 1999; Magrane and Manfredi, 2009; Rothstein, 2009) and might contribute to the triggering of motor neuron death. Additional novelty of the present analysis was the demonstration of deregulated transcripts-related to cytoskeleton by DAVID categorization in the sciatic nerve of the presymptomatic ALS mice. Gene profiling in the sciatic nerve accounts for axonal (minority) and Schwann cell (majority) transcripts (Baraban et al., 2013; Malmqvist et al., 2014), thus adding an important contribution to molecular analysis on dying back hypothesis of ALS pathology (Dadon-Nachum et al., 2011; De Oliveira et al., 2014).

Despite the evidence of protein synthesis in growing or regenerating axons *in vitro* and of mRNA axonal transport and local translation in developing zebrafish (Baraban et al., 2013), it is still not clear whether axons of adult motor neurons contain ribosomes and other elements that are necessary for protein translation (Jablonka et al., 2004). Altogether, this manuscript highlights the importance to evaluate the cytoskeleton changes in the Schwann cells in the still poorly described peripheral pathology in ALS (Xiao et al., 2006) and their contribution to impair paracrine trophic actions to motor neurons (unpublished results from our laboratory, presented as an abstract form in the 2013 Society for Neuroscience Meting, San Diego, USA). In order to address this issue, we have developed and presented here different methods to enrich specific cell types for molecular analysis.

### Deregulated genes of cytoskeleton molecules already pointed in ALS

The description of 14 differentially expressed genes (six down and eight upregulated) in the spinal cord of 40 days old presymptomatic ALS mice already indicates a very early presymptomatic event related to cytoskeleton with a possible implication to physiopathological mechanisms of the disease onset. From those genes, only *Kif3a* and *Kif1b* or related molecules have been studied in the context of kinesin dysfunction or impaired anterograde transport of cargos, like neurofilament, in ALS (Dupuis et al., 2000; Conforti et al., 2003; Pantelidou et al., 2007). The *Kif3a* (downregulation) and *Kif1b* (upregulation) deregulated genes seen in the spinal cord of 40 days old SOD1^G93A^ mice were in line with descriptions of reduction KIF3Aβ in motor cortices of ALS human and animal model (Pantelidou et al., 2007) and of KIF3-associated proteins in ALS rodents (Dupuis et al., 2000), thus underlining the presence of an early and complex mechanism involved in the impairment of the fast anterograde axonal transport machinery in ALS prior motor neuron degeneration.

From the 76 deregulated genes (25 down and 51 upregulated) in the spinal cord of 80 days old presymptomatic ALS mice, only eight genes (*Actg1*, *Adora*, *Akt1*, *App*, *Dctn1*, *Kif1a*, *Sirt2*, and *Stmn1*) or related molecules have been studied in the context of ALS.

The upregulation of *Kif1a* is in accordance to previous description (Dupuis et al., 2000) and might represent a regulatory mechanism in order to compensate the impaired anterograde transport in neurons. KIF1 is divided into KIF1A, responsible for transport of synaptic vesicle precursors (Okada et al., 1995), and KIF1B, described above, a monomeric motor responsible for the anterograde transport of mitochondria (Nangaku et al., 1994).

*Actg1* deregulation described in the spinal cord of 80 days old ALS mice was already mentioned in a previous ALS publication (Baciu et al., 2012) and the related actin product of the gene might impair dendritic spine plasticity with potential implication to motor neuron toxicity in ALS (Sunico et al., 2011). Such a mechanism may also involve an impairment of purinergic receptor-mediated actin cytoskeleton remodeling (Goldman et al., 2013), which is in line with the upregulation of *Adora1* transcription codifying adenosine A1 receptor, described in this and previous studies (Gundlach et al., 1990).

*Dctn1* expression was upregulated in presymptomatic spinal cord (80 days old) of ALS mouse model, thus denoting dynactin impairment as a mechanism in the presymptomatic phase of the disease. Mutation in *Dctn1* gene has been associated to motor neuron degeneration in ALS (Hafezparast et al., 2003) and downregulation of the gene was described in residual motor neurons of postmortem material of ALS patients (Jiang et al., 2007). There is a lack of information on dynactin regulation before clinical onset of ALS despite the fact that dynein-dynactin complex, the only retrograde transport motor, contributes to formation of SOD1 inclusions in the disease (Strom et al., 2008). Furthermore, deregulation of Schwann cell genes related to neurothropin-dependent mechanisms in association to an impaired axonal transport may enhance motor neuron vulnerability in ALS (Koh et al., 2005; Niewiadomska et al., 2011).

The upregulation of *Sirt2* expression in the spinal cord of 80 days old mice might trigger toxicicity to motor neurons in the late stage of the presymptomatic age by increasing deacetylation of alfa tubulin (Korner et al., 2013; Taes et al., 2013). Moreover, the upregulation of *Stmn1* in the ALS spinal cord before neuronal death is in agreement to previous description on stathmin protein accumulation in spinal cord motor neurons leading to Gogi apparatus fragmentation and collapse of microtubule network (Strey et al., 2004). Furthermore, the formation of perikaryal/axonal intermediate filament inclusions, neurofilament abnormalities and genetic defects in microtubule-based transport that may facilitate the elevation of the toxic amyloid beta precursor in ALS (Spadoni et al., 2009; Bryson et al., 2012) might correlate the downregulation of *App* seen in the spinal cord of 80 days presymptomatic phase to a neuroprotective regulation before the neuronal death onset.

Finally, the downregulation of *Vapa* in the spinal cord of 80 days old ALS mice is also an original and interesting finding of the present analysis. VAMP/synaptobrevin-associated proteins A and B (VAPA and VAPB) are both enriched on endoplasmic reticulum and Golgi membranes and are capable to interact with cytoskeleton elements in order to maintain the organelle morphology (Nishimura et al., 1999). Despite a lack of information on VAPA function in the central nervous system, VAPB mutation is associated to a familial form of ALS (Nishimura et al., 2004).

It should be pointed out the regulation *Maea*, the unique gene that was overexpressed in the spinal cord of both 40 and 80 days old ALS mice, indicating its long lasting involvement in the presymptomatic events in the ALS spinal cord. The role of *Maea* in ALS is unknown but it could participate in the immunomodulatory signaling of non-neuronal cells-induced toxicity in ALS (Levine et al., 1999; McGeer and McGeer, 2002; Pasinelli and Brown, 2006).

From the 146 deregulated genes (74 down and 72 upregulated) in the sciatic nerve of 60 days presymptomatic old SOD1^G93A^ old mice, only 10 genes or related molecules have been studied in context of ALS.

The downregulation of the *Aif1*, *Ccnb1*, and *Mapt* in the presymptomatic ALS mice is likely to participate in the early events in the ALS peripheral nerve pathology. *Aif1* encodes the allograft inflammatory factor-1 (AIF-1) and AIF-1 positive microglia/macrophages are among the earliest cells to respond to nerve injury (Schwab et al., 2001). It is likely that AIF-1 may act as an initiator of the early microglial/macrophage-induced immunomodulation leading a motor axon retraction and neuromuscular junction disruption before neuronal degeneration (Dibaj et al., 2011). Furthermore, *Ccnb1* deregulation-induced cytoskeleton disorganization (Husseman et al., 2000) and also altered neuronal cytoskeleton protein Tau encoded by *Mapt* -induced microtubule stabilization and assembly deregulation (Aronov et al., 2002) are possible mechanisms related to early axonal retraction taking place in presymptomatic phases of the disease (Aronov et al., 2002).

*Actn3*, *Als2*, *Kif5a*, *Kif5c*, *Nos3*, and *Tmod3* were found upregulated in the sciatic nerve of 60 days old presymptomatic ALS mice and their related molecules have been mentioned in the context of ALS mechanisms. ACTN3, one of the four human alpha-actinin isoforms, has been associated to ALS progression in human muscle (Pradat et al., 2012; Bernardini et al., 2013). Moreover, the upregulated *Tmod3*, which codifies the tropomodulin (TMOD), an actin-capping protein for the slow-growing end of filamentous actin (Ito et al., 1995), may represent a need for the dynamic polymerization of actin cytoskeleton, probably in the Schwann cells of ALS nerve. Interestingly, the mutation of ubiquitously expressed TMOD3 protein is responsible for type 5 familial ALS (Cox and Zoghbi, 2000). Furthermore, despite ALS2 deficiency accounts for ALS2 familial form (Hadano et al., 2001; Yang et al., 2001), the upregulation of *Als2* seen in the 60 days old presymptomatic ALS sciatic nerve could reflect a transient Schwann cell neuroprotective paracrine response (Hadano et al., 2010). In fact, ALS2/alsin, a guanine nucleotide exchange factor for GTPase Rab5, is involved in endosome fusion/trafficking, neurite outgrowth and corticospinal axon integrity (Deng et al., 2007; Lai et al., 2009), probably by interfering with the accumulation of immature vesicles and misfolded proteins (Lai et al., 2009).

We have also found a deregulation of the *Nos2* and *Nos3* expression in sciatic nerve of 60 days old ALS mice, without changes in presymptomatic spinal cord. The synthesis of inducible NOS in the spinal cord and peripheral nerve of ALS model in the presymptomatic phase of the disease has been mentioned (Almer et al., 1999; Chen et al., 2010).

Important finding of the present study was also the regulation of several genes of kinesin molecules in the sciatic nerve of 60 days old presymptomatic ALS mice. The differential *Kif5* regulation in the sciatic nerve observed in our microarray analysis may in fact represent the gene regulation in the Schwann cells where the KIF5 participate in the myelin integrity (Bolis et al., 2009). In this context, KIF5B might be of substantial interest because it also expresses in non-neuronal cells and its regulation/activity has not been explored in ALS mechanisms. Further evidence for KIF5 mechanisms in non-neuronal cells were obtained from the absence of *Kif5* expression in the spinal cord of presymptomatic SOD1^G93A^ mice by gene profiling study (this work) and qPCR analysis (Kuzma-Kozakiewicz et al., 2013).

### Topographic and cellular modulation of *Kif1b* of kinesin family

The downregulation of *Kif1b* in the sciatic nerve showed by microarray and qPCR was in the opposite direction to the upregulation of the gene seen in the spinal cord of presymptomatic ALS mice. Importantly, only the *Kif1b* was deregulated in the two evaluated regions and also was reported previously in the context of ALS (Ferraiuolo et al., 2007; Kuzma-Kozakiewicz et al., 2013). Furthermore, it is the first time *Kif1b* expression or its protein has been described in Schwann cells. The development of technology to enrich Schwann cells allowed the present analysis.

*Kif1b* deregulation in presymptomatic ALS seems to be an important event, specially in the Schwann cells because KIF1B is required for adequate myelination process by oligodendrocytes (Lyons et al., 2009). Presently, myelin pathology is not clear in ALS peripheral nerves (Heads et al., 1991). Nonetheless, it is still undefined whether peripheral myelin morphological alteration in ALS is a consequence of axonal degeneration (Perrie et al., 1993).

There is a lack of information on myelin alterations in peripheral nerves of presymptomatic ALS mice and it is uncertain whether impairments in the Schwann cell function could contribute to ALS axonal pathology and dying back events. That is actually an important issue in the pathogenic mechanism of the disease because myelin cell function overtakes action potential conduction along peripheral axons (Monk and Talbot, 2009). In fact, the control of axoplasmic Ca^2+^ and posttranslational modifications of local trafficking proteins are part of trophic support signaling provided by myelinating cells. We might speculate that an impaired crosstalk between Schwann cell and motor axon in presymptomatic stages of the disease could trigger axonal retraction and Wallerian degeneration (Lyons et al., 2009; Kiryu-Seo and Kiyama, 2011; Gentil and Cooper, 2012).

The above discussion indicates the importance to evaluate the events in specific cell types notably in the context of peripheral nerve-induced neuropathology in ALS, because a differential regulation can occur specifically in Schwann cells and also altered axonal transport might modify the local traffic of RNAs (Ticozzi et al., 2010).

Single cell laser microdissection has been employed by our group and other researchers to evaluate gene expression on enriched cell types (Ferraiuolo et al., 2007; De Oliveira et al., 2009; Guipponi et al., 2010; Kudo et al., 2010; Tanaka et al., 2012). We have developed and showed here for the first time the method to immunolabel mouse Schwann cells and motor neurons and also the procedures to enrich cell samples for molecular analyses by means of single cell laser microdissection. We have also developed and demonstrated in this work the methodology to enrich mouse Schwann cells by means of primary cell culture and cell sorting. We still do not know precisely the limitations of the enrichment techniques employed here. Nevertheless, it should be emphasized the agreement of the results obtained by the different methods.

It is of substantial importance that the methodology allowed the observation of a differential regulation of *Kif1B* in the enriched ChAT immunolabeled motor neurons (upregulation) and Schwann cells (downregulation). The results were coincident to those obtained in qPCR and microarray analyses of whole spinal cord and sciatic nerve of presymptomatic ALS mice.

A differential gene regulation in specific cell types in a neuroglial unit, the motor neuron-Schwann cell unit in this case, highlights the complexity of cellular and molecular mechanisms of ALS, remarkably before clinical onset. *Kif1b* upregulation in immunolabeled motor neurons was in line to a previous work that employed toluidine blue-enriched putative motor neurons of presymptomatic ALS mice (Ferraiuolo et al., 2007). This finding indicates an involvement of a motor protein of kinesin family in the axonal trafficking before the death of motor neurons and the appearance of neurological symptoms. The elevation of the protein in ALS neurons might be a substrate for an increased kinesin-1 phosphorylation and a diminution of kinesin-1 function with a subsequent defect of fast axonal transport (Morfini et al., 2013).

The *Kif1b* downregulation in the sciatic nerve and also in the enriched Schwann cells of presymptomatic ALS mice is a major original contribution of the present work. The recent description on the role of KIF1B for the adequate function of central myelinating cells (Lyons et al., 2009; Gentil and Cooper, 2012) opens up the possibly for the existence of KIF1B mechanisms in the paracrine trophic actions of Schwann cells to peripheral motor neurons. Deregulated KIF1B in Schwann cells highlights the possibility of no autonomous cell toxicity of Schwann cells to motor neurons in ALS, mechanisms that should be investigated in details in future works. In fact, the non-autonomous cell toxicity of central glia to motor neurons has been described (Boillee et al., 2006a) and the related molecular pathways are under investigation.

In conclusion, the present work demonstrated cytoskeleton gene regulation as an important occurrence in motor neurons and Schwann cells in the presymptomatic stages of ALS and may be of importance in the dying back mechanisms of neuronal death in the neurodegenerative disease. The differential regulation of *Kif1b* in the spinal cord (upregulation) and sciatic nerve (downregulation) was coincident to that found in the enriched motor neurons and Schwann cells and emerged as an important event in the pathogenic mechanism of ALS.

## Author contributions

Jessica R. Maximino, Gabriela P. de Oliveira, and Chrystian J. Alves performed the experiments. Jessica R. Maximino and Gerson Chadi designed the study and analyzed the results. Gerson Chadi wrote the manuscript. All authors read and approved the final manuscript.

### Conflict of interest statement

The authors declare that the research was conducted in the absence of any commercial or financial relationships that could be construed as a potential conflict of interest.
