# Supplementary material for: Deregulated expression of cytoskeleton related genes in the spinal cord and sciatic nerve of presymptomatic SOD1G93A Amyotrophic Lateral Sclerosis mouse model
Source: Front Cell Neurosci. 2014 May 26;8:148. doi: 10.3389/fncel.2014.00148 (PMC4033281; doi:10.3389/fncel.2014.00148)
Supplement: Supplementary file 1 [file DataSheet1.PDF]

Supplementary Material for:
Deregulated expression of cytoskeleton related genes in the spinal cord and sciatic nerve of $SOD1_{G93A}$ Amyotrophic Lateral Sclerosis mouse model

Jessica Ruivo Maximino, Gabriela Pintar de Oliveira, Chrystian Junqueira Alves, Gerson Chadi*

Neuroregeneration Center, Department of Neurology, University of São Paulo School of Medicine, São Paulo, Brazil, 01246-903

*Corresponding author:
Gerson Chadi, M.D., Ph.D.
Full Professor
Department of Neurology
University of São Paulo
Av. Dr. Arnaldo, 455, 2nd floor, room 2119
01246-903, São Paulo. Brazil
Phone: 55 11 3061-7460
gerchadi@usp.br

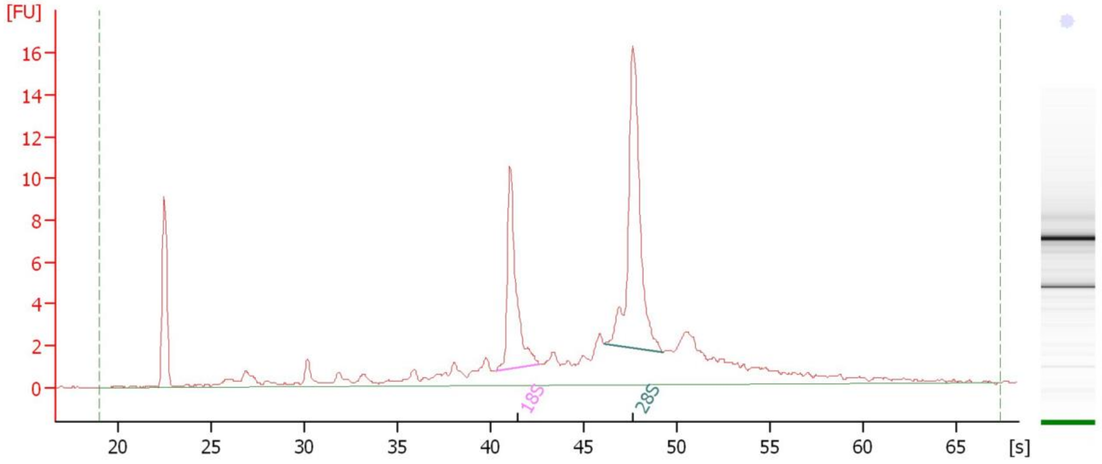

Figure S1. Electropherogram representative of the results of RNA integrity obtained from samples employed in the experiments. The RNA integrity calculation is based on the ratios between 28S and 18S subunities from ribosomal RNA. The sample chosen for illustration ($SOD1^{G93A}$ mouse sciatic nerve) presented a RNA integrity number (RIN) equal 8, once values of all samples ranged from 7.0 to 8.9.

Demonstration of motor neuron enrichment by PCR

Laser microdissected mouse motor neuron samples were submitted to PCRs to certify sample purity. Primers to evaluate the presence of motor neuron, astrocyte and microglia, respectively, *Chat*, *Gfap* and *Cd68*, are shown in Table. The reactions were performed as described bellow. Whole spinal cord sample was used as a positive control. PCR products were submitted to electrophoresis as described bellow and the result is shown in Figure S2.

Sequence for primers to evaluated motor neuron enrichment.

GeneID	Primer 5'-3'	Amplicon (bp)
Chat	F: CAAATAAGTCATAAAGGCAGAGGC	140
	R: CTCAAGGAAGACTGTGCTATGG	
Gfap	F: CAGACTTTCTCCAACCTCCAG	138
	R: CTCCTGCTTCGAGTCCTTAATG	
Cd68	F: ACTTCGGGCCATGTTTCTC	136
	R: TGGTAGGTTGATTGTCGTCTG	

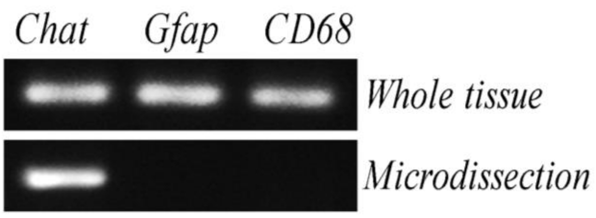

Figure S2. PCR results for mouse motor neuron sample enrichment. Representative bands of PCRs for specific gene markers of motor neurons (*Chat*), astrocytes (*Gfap*), and microglia (*Cd68*) in whole lumbar spinal cord and in the motor neuron enriched samples. Only *Chat* band could be amplified by PCR, which strongly indicates a high level of motor neuron enrichment in the samples.

Demonstration of Schwann cell enrichment by PCR

Schwann cells obtained by laser microdissection, fluorescence activated cell sorting and cell culture were submitted to PCRs for sample purity verification. Primers to evaluate the presence of Schwann cells (*S100)* and fibroblast (*Thy1)* in the PCRs are shown in Table as well as to the internal control *Gapdh*. The reactions were performed to 20µl final volume, using GoTaq Flexi DNA Polymerase (Promega), according to manufacturers and 500nM of each primer. The protocol for PCRs consisted in 95°C during 5 minutes, followed by 35 cycles of 95°C during 30 seconds, 60°C during 30 seconds, 72°C during 45 seconds, ending with 72°C in 7 minutes. Whole sciatic nerve sample was used as a positive control. PCR products were submitted to electrophoresis in 2% agarosis gel containing ethidium bromide for 60 minutes at 100V, and then visualized under UV exposure. The results are shown in Figure S3.

Sequence for primers to evaluated Schwann cell enrichment.

GeneID	Primer 5'-3'	Amplicon (bp)
S100	F: CCCTCATTGATGTCTTCCACC	150
	R: TCTCCATCACTTTGTCCACC	
Thy1	F: GTCCTTACCCTAGCCAACTTC	134
	R: CCGCCACACTTGACCAG	
Gapdh	F: GAGTAAGAAACCCTGGACCAC	111
	R: TCTGGGATGGAAATTGTGAGG	

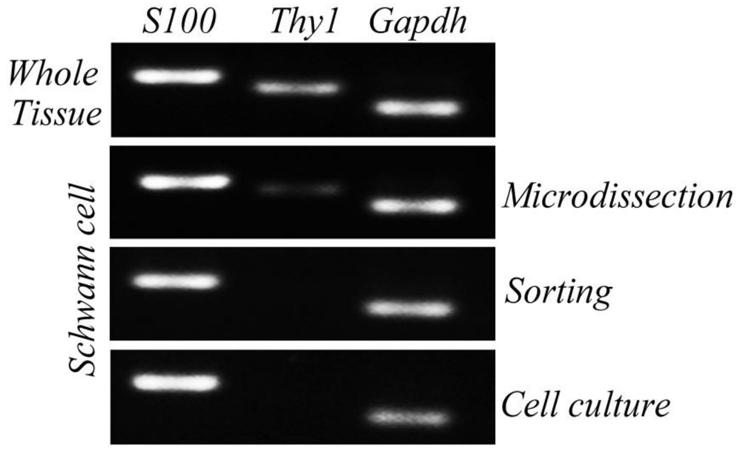

Figure S3. PCR results for Schwann cell sample enrichment of mice. Representative bands of PCRs for specific gene markers of Schwann cells (*S100*), fibroblasts (*Thy1*), and *Gapdh* in Schwann cells-enriched samples obtained by laser microdissection, flow cytometry sorting and cell culture after purification. Mouse whole sciatic nerve sample was used as a positive control.

Demonstration of Schwann Cell Enrichment by Flow Cytometry

Samples of Flow Cytometry Sorting procedures of $SOD1^{G93A}$ and wild-type mice were submitted to a double immunolabeling to identify Schwann cells and fibroblasts by means of p75NGF receptor cell surface labeling as described in the text and a fluorescein phychoerythrin (PE-Cy5)-conjugated monoclonal antibody against Thy-1 (Abcam, USA), respectively. The cells were analyzed on a FACSAria III Cell Sorter (BD Biosciences, USA) as described in the text. A dot plot of the data showed two distinct cell populations upon specific light scattering by flow cytometry analysis (Figure S4).

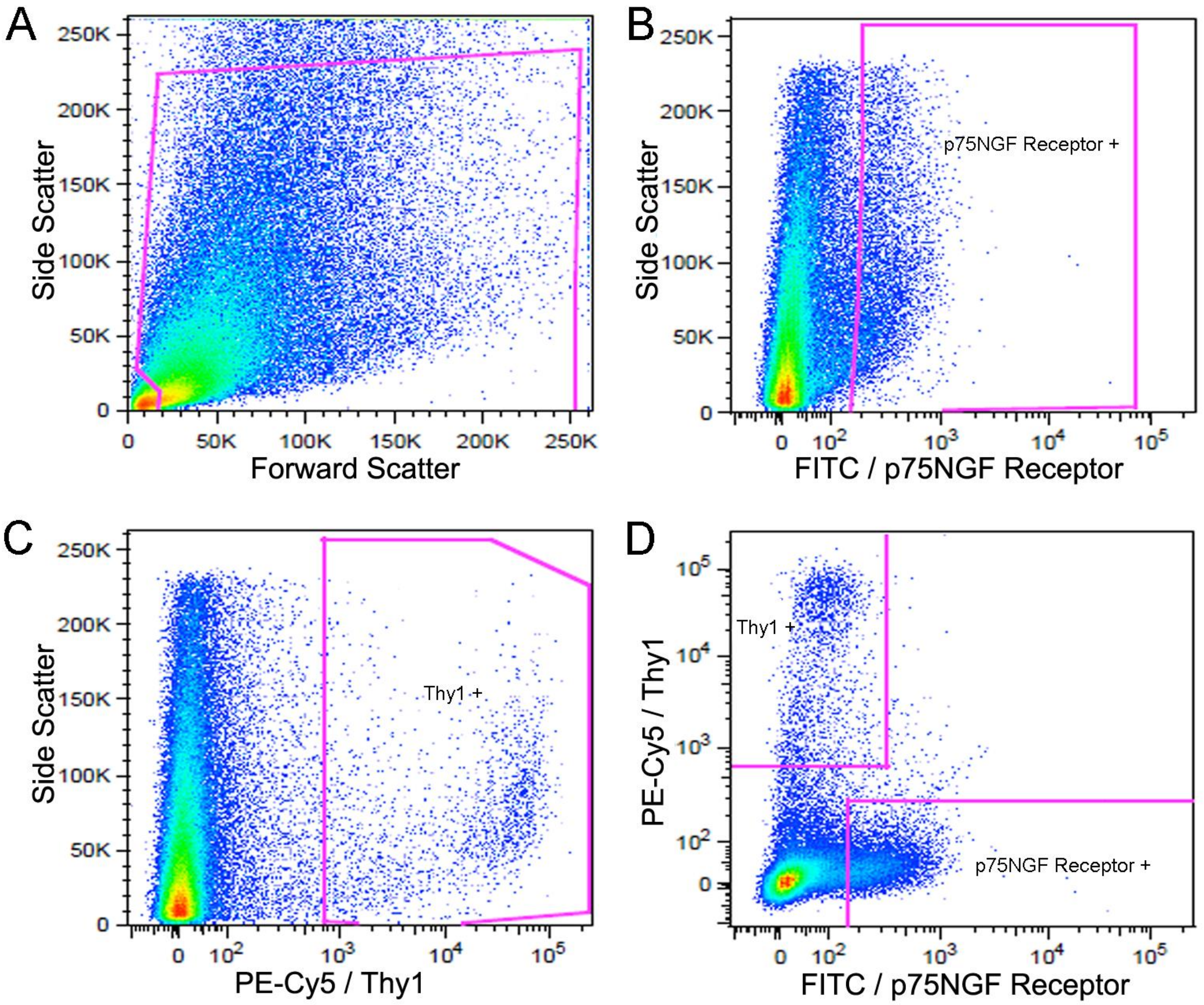

Figure S4. Flow cytometry analysis of Schwann cells and fibroblasts from a mouse sciatic nerve employed in the experiments. FITC-conjugated p75NGF receptor and PE-Cy5-conjugated Thy1 antibodies were employed in the two-color immunolabeling of Schwann cells and fibroblasts, respectively (A-D). Dot plots indicate the total number of events in the sciatic nerve cell suspension and the dots inside the red box represent the excluded doublet and dead profiles, which have been eliminated by morphological criteria according to previous descriptions (Shapiro, 2005; Herzenberg et al., 2006) (A). After morphological criteria, dot plots of Schwann cell (B) and fibroblast (C) profiles were obtained using respective fluorescence filters and the blots inside the red boxes represent the specific profiles after discounting the unspecific labeling. Positive p75NGF receptor and Thy1 cell profiles are shown in corresponding boxes after FITC *vs* PE-Cy5 fluorescence intensity plotting (D).
